# Pediatric Rosai-Dorfman-Destombes Disease: From Isolated Cervical Lymphadenopathy to Multisystem Disease

**DOI:** 10.7759/cureus.116260

**Published:** 2026-09-15

**Authors:** Soufia Charaf, Sanaa Bouramdane, Kenza Elmkaddem, Nawal Hammas, Meryem Boubbou, Sarra Benmiloud

**Affiliations:** 1 Department of Pediatric Hematology-Oncology, Hassan II University Hospital, Sidi Mohamed Ben Abdellah University, Fez, MAR; 2 Department of Pathology, Hassan II University Hospital, Sidi Mohamed Ben Abdellah University, Fez, MAR; 3 Department of Mother and Child Radiology, Hassan II University Hospital, Sidi Mohamed Ben Abdellah University, Fez, MAR

**Keywords:** cervical lymphadenopathy, emperipolesis, non-langerhans cell histiocytosis, pediatric histiocytosis, rosai-dorfman-destombes disease

## Abstract

Rosai-Dorfman-Destombes disease (RDD) is a rare non-Langerhans cell histiocytosis with a broad clinical spectrum, ranging from localized lymphadenopathy to severe multisystem involvement. Due to its rarity, clinical data on pediatric RDD remain limited, particularly in North Africa. We report two contrasting pediatric cases of RDD managed at a tertiary center in Morocco to illustrate this clinical heterogeneity. The first was that of a five-year-old girl who presented with chronic, isolated, painless cervical lymphadenopathy. Laboratory findings were normal. Biopsy confirmed RDD with classic emperipolesis and an S100+, CD163+, CD68+, CD1a− immunophenotype. Targeted molecular analysis showed no mutations in *KRAS*, *NRAS*, or *BRAF* exon 15. Active surveillance led to progressive spontaneous regression. The second case was of a 20-month-old boy initially misdiagnosed with osteomyelitis due to painful bone lesions. He rapidly developed severe multisystem disease with axillary lymphadenopathy, respiratory distress secondary to pulmonary nodules, hepatomegaly, renal lesions, and widespread osteolytic destruction. Laboratory testing revealed a marked inflammatory syndrome, anemia, and polyclonal hypergammaglobulinemia. Subsequent lymph node biopsy established the diagnosis of RDD. Treatment with vinblastine and prednisone achieved long-term complete remission, with persistent bone deformity as the sole sequela. Pediatric RDD poses a significant diagnostic challenge due to its marked clinical variability. Histopathological confirmation showing emperipolesis and characteristic immunophenotyping is essential. Isolated nodal forms carry a favorable prognosis and can be managed conservatively, whereas severe multisystem disease requires prompt systemic therapy.

## Introduction

Rosai-Dorfman-Destombes disease (RDD), or sinus histiocytosis with massive lymphadenopathy, is a rare non-Langerhans cell histiocytic disorder characterized by histiocytic accumulation within lymph node sinuses and extranodal tissues [1,2]. Although first described in 1965, its pathogenesis remains partially understood, involving potential viral triggers (Epstein-Barr virus (EBV), human herpesvirus (HHV-6)), immune dysregulation, or somatic mitogen-activated protein kinase (MAPK)/extracellular signal-regulated kinase (ERK) pathway mutations [1,2].

Clinically, RDD typically presents with massive, painless cervical lymphadenopathy, though extranodal involvement occurs in 40-45% of cases [1-3]. Because its clinical and radiological features frequently mimic infectious, autoimmune, or malignant processes, definitive diagnosis is based on histopathology showing emperipolesis, along with S100, CD68, and CD163 positivity and CD1a negativity [1,2,4].

Importantly, published pediatric data remain insufficient, particularly in low- and middle-resource settings such as Morocco, where diagnostic delays occur, and regional literature is sparse [5-8]. Management is further complicated by the lack of standardized guidelines, requiring tailored strategies ranging from observation to surgery, corticosteroids, or systemic therapies [2].

To address this gap, we describe the clinical, pathological, therapeutic, and outcome characteristics of two pediatric RDD cases from a Moroccan tertiary referral center, highlighting the diagnostic difficulties and therapeutic choices associated with this rare disorder in children.

## Case presentation

Case 1

A five-year-old girl with no significant past medical history presented with a one-year history of a progressively enlarging left cervical mass. On initial examination, the patient was afebrile, had no night sweats, and maintained a good general condition. Physical examination revealed bilateral painless cervical lymphadenopathy, measuring less than 1 cm on the right and approximately 3 cm on the left, without overlying skin inflammation or clinical features of extranodal involvement. Initial laboratory investigations, including complete blood count (CBC), erythrocyte sedimentation rate (ESR), serum protein electrophoresis, and infectious and autoimmune screening, were within normal parameters.

Cervical ultrasonography demonstrated a conglomerate mass comprised of multiple left cervical lymph nodes measuring 3.5 × 2 cm (largest node: 2.1 × 1.9 cm) without evidence of necrosis. Definitive diagnosis of RDD was established via excisional lymph node biopsy, which demonstrated preserved nodal architecture, sinus histiocytosis, and prominent emperipolesis within large eosinophilic histiocytes (Figure 1, Figure 2). Immunohistochemical staining confirmed positivity for S100, CD68, and CD163 and negativity for CD1a, excluding Langerhans cell histiocytosis. Staging cervico-thoraco-abdomino-pelvic computed tomography (CT) scan showed no evidence of extranodal or systemic involvement, and molecular analysis revealed no mutations in the examined exons of *KRAS*, *NRAS*, or *BRAF* exon 15.

**Figure 1:**
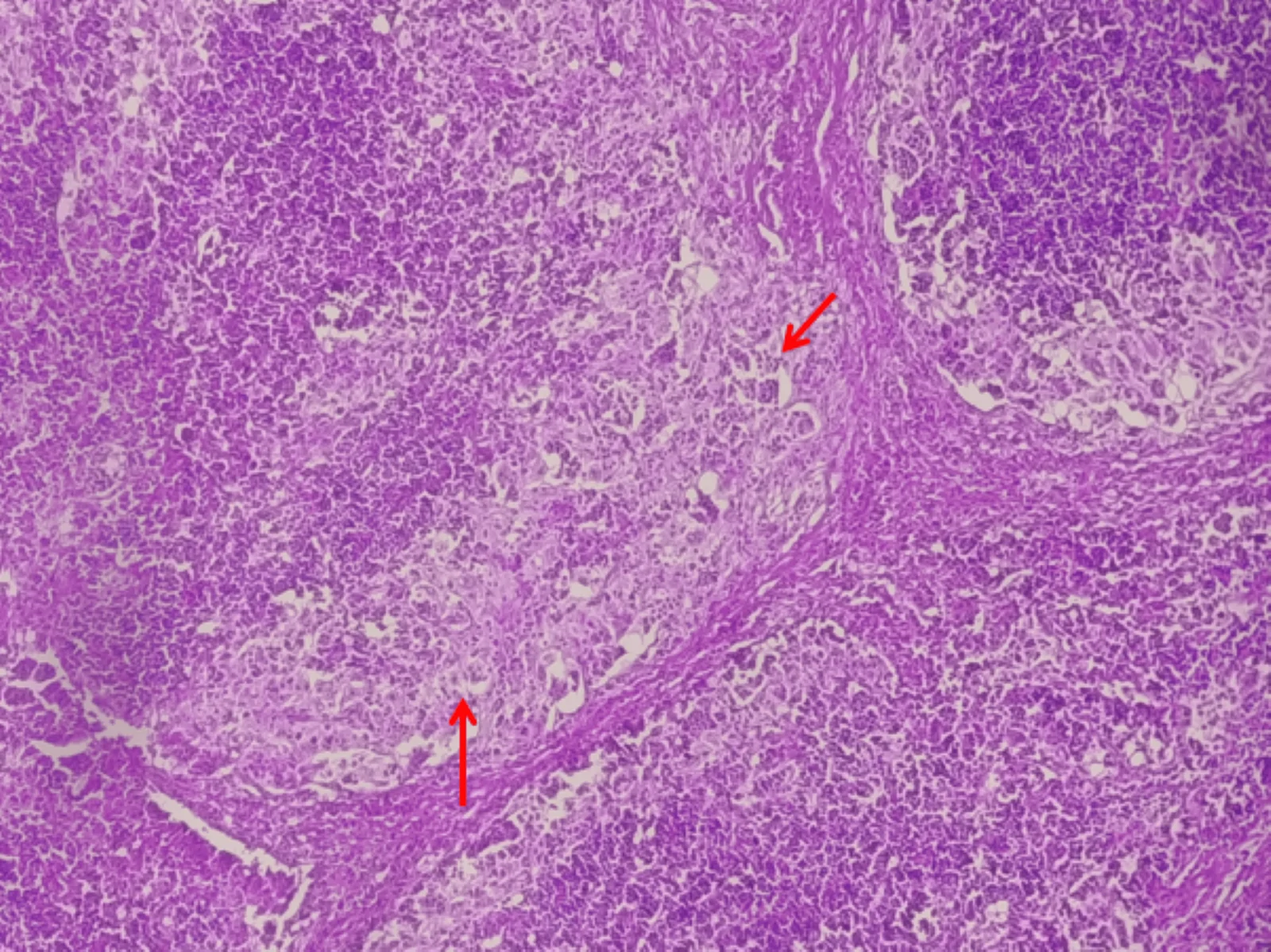
Histopathological examination of the excised lymph node (HES, ×100) showing expanded interfollicular sinusoids with diffuse histiocytic infiltration and partial effacement of the nodal architecture (red arrows). HES: hematoxylin-eosin-saffron

**Figure 2:**
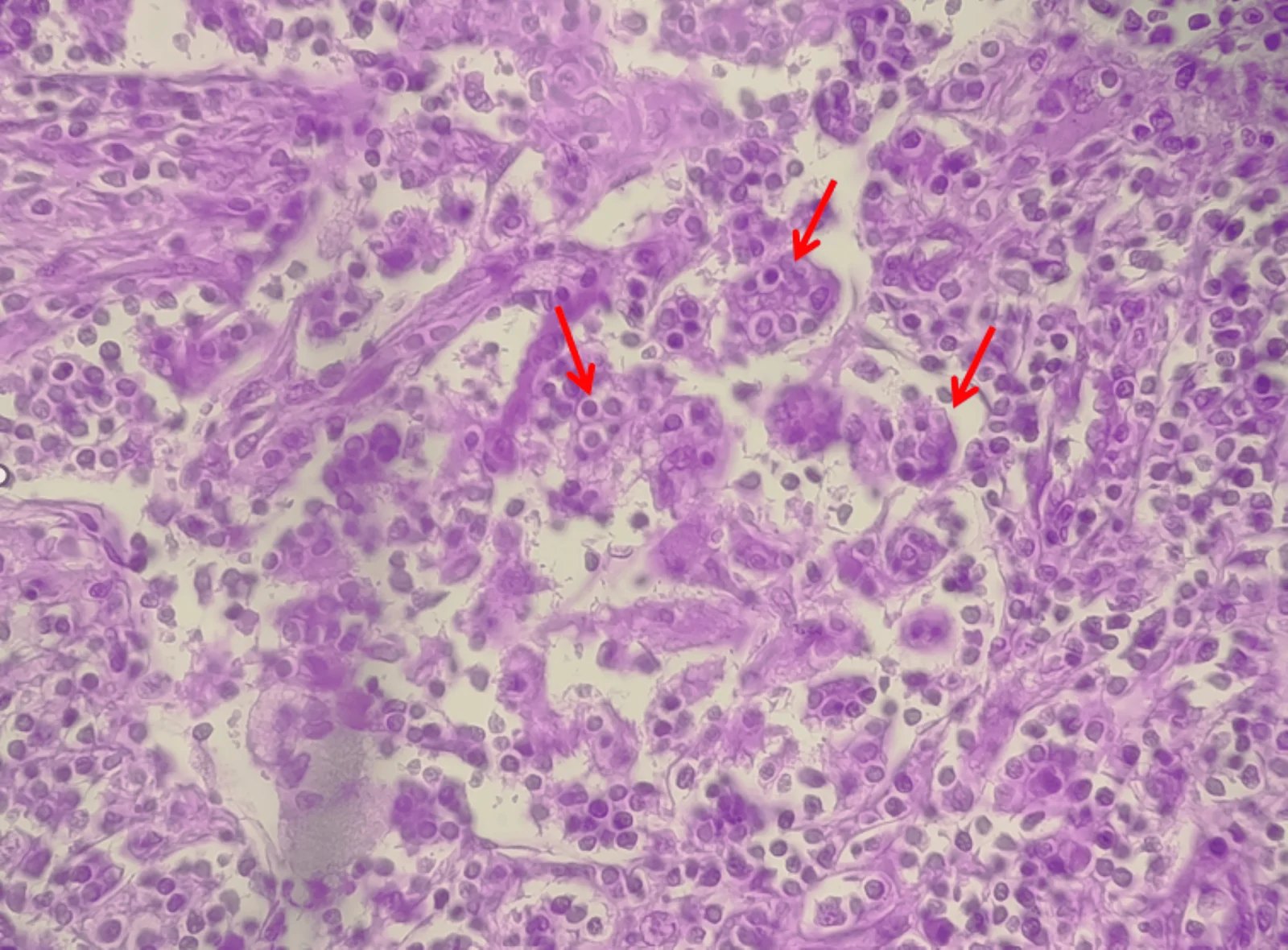
Histopathological examination of the excised lymph node (HES, ×400) demonstrating large histiocytes with emperipolesis (arrows). HES: hematoxylin-eosin-saffron

Given the localized nodal disease, absence of systemic involvement, and favorable clinical status, a conservative approach with clinical and radiological surveillance every three months was adopted. Progressive regression of the lymphadenopathy was observed, becoming subcentimetric at six-month follow-up. At two-year follow-up, the patient remained clinically asymptomatic, with stable radiological findings and no evidence of disease progression or recurrence.

Case 2

This case was previously reported as an individual case because of its unusual bone involvement [7]. The earlier single-case publication had simplified the clinical trajectory by attributing the diagnosis directly to the bone biopsy. However, here, our objective is to reflect the diagnostic journey and highlight the significant challenge of diagnosing extra-nodal pediatric RDD when initial osseous biopsies present as non-specific reactive lesions.

A 20-month-old boy presented with painful swelling of the left forearm associated with complete functional impairment following minor trauma three months earlier. Initial physical examination revealed an afebrile infant in good general condition, with painful swelling of the left forearm and functional limitation, without clinical evidence of systemic involvement. Initial laboratory investigations revealed neutrophilic leukocytosis of 17,500/µL, elevated C-reactive protein (CRP) of 125 mg/L, and an elevated ESR of 60 mm at the first hour and 80 mm at the second hour. Standard radiography of the left upper limb showed aggressive osteolytic lesions of the radial diaphysis with cortical destruction and periosteal reaction. Ultrasonography revealed a large juxtacortical collection suggestive of osteomyelitis with a subperiosteal abscess. Based on these findings, an initial presumed diagnosis of osteomyelitis complicated by subperiosteal abscess was considered. Empirical antibiotic therapy was initiated, and surgical drainage with bone biopsy was performed.

Histopathological examination of the initial bone biopsy revealed a pseudotumoral histiocytic reaction, initially interpreted as reactive to osteitic lesions. Immunohistochemistry showed positivity for CD45 and S100 proteins and negativity for CD1a. However, due to this initial interpretation, the diagnosis of RDD was established only after subsequent clinicopathological reassessment prompted by disease progression and lymph node biopsy.

After two weeks, symptoms persisted, with the onset of respiratory distress, hepatomegaly, and development of a firm, fixed, painless, non-inflammatory left axillary lymphadenopathy measuring approximately 2 cm. Standard radiography of the left forearm showed extensive destruction of the left radius (Figure 3, Figure 4), residual bone fragments, and development of a soft tissue mass centered on the radius, associated with soft tissue thickening and calcifications, with a second osteolytic lesion of the medial humeral condyle (Figure 5). Laboratory evaluation showed normochromic microcytic anemia with a hemoglobin level of 8 g/dL, neutrophilic leukocytosis at 20,500/µL, and a marked inflammatory syndrome, with an ESR of 101 mm at the first hour and 120 mm at the second hour. Serum protein electrophoresis showed hypoalbuminemia with elevated alpha-1, alpha-2, and gamma globulin fractions, consistent with chronic inflammation.

**Figure 3:**
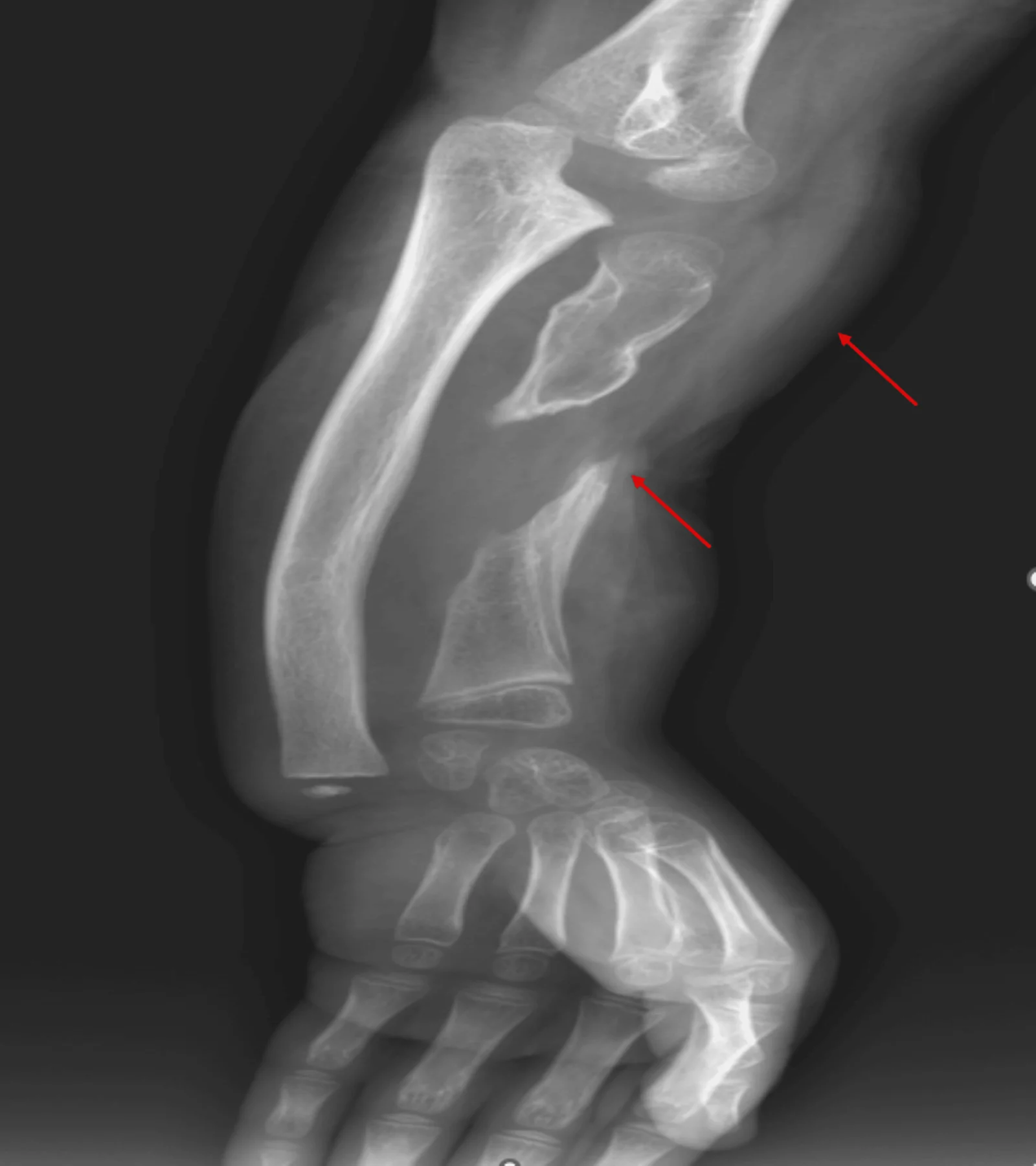
Frontal radiograph of the left forearm demonstrating severe cortical breach with extensive osteolytic destruction of the radial shaft (lower red arrow), alongside an associated adjacent soft tissue mass (upper red arrow).

**Figure 4:**
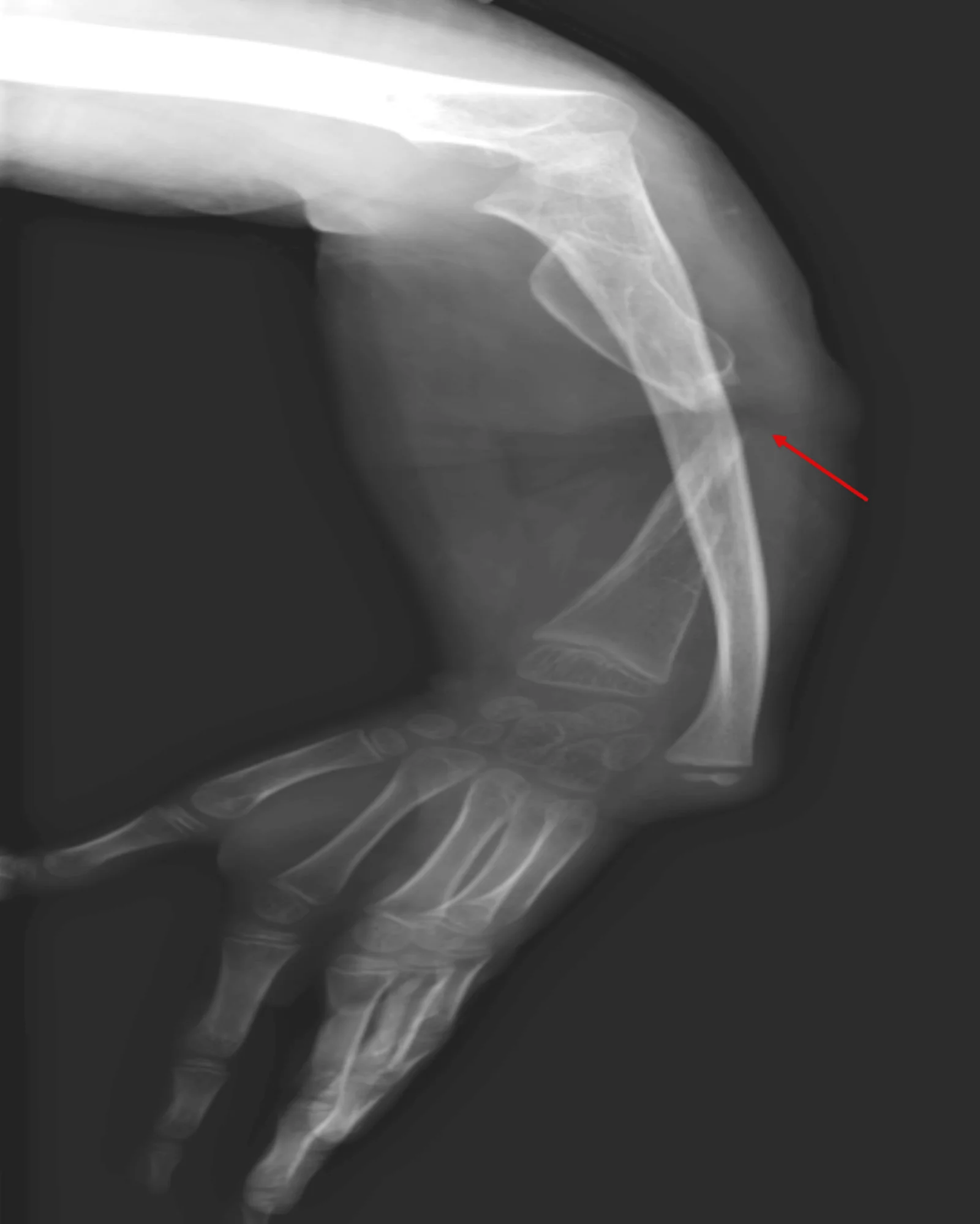
Lateral radiograph of the left forearm showing extensive radial diaphysis destruction, pathological displacement, and surrounding soft tissue expansion centered on the bone defect (red arrow).

**Figure 5:**
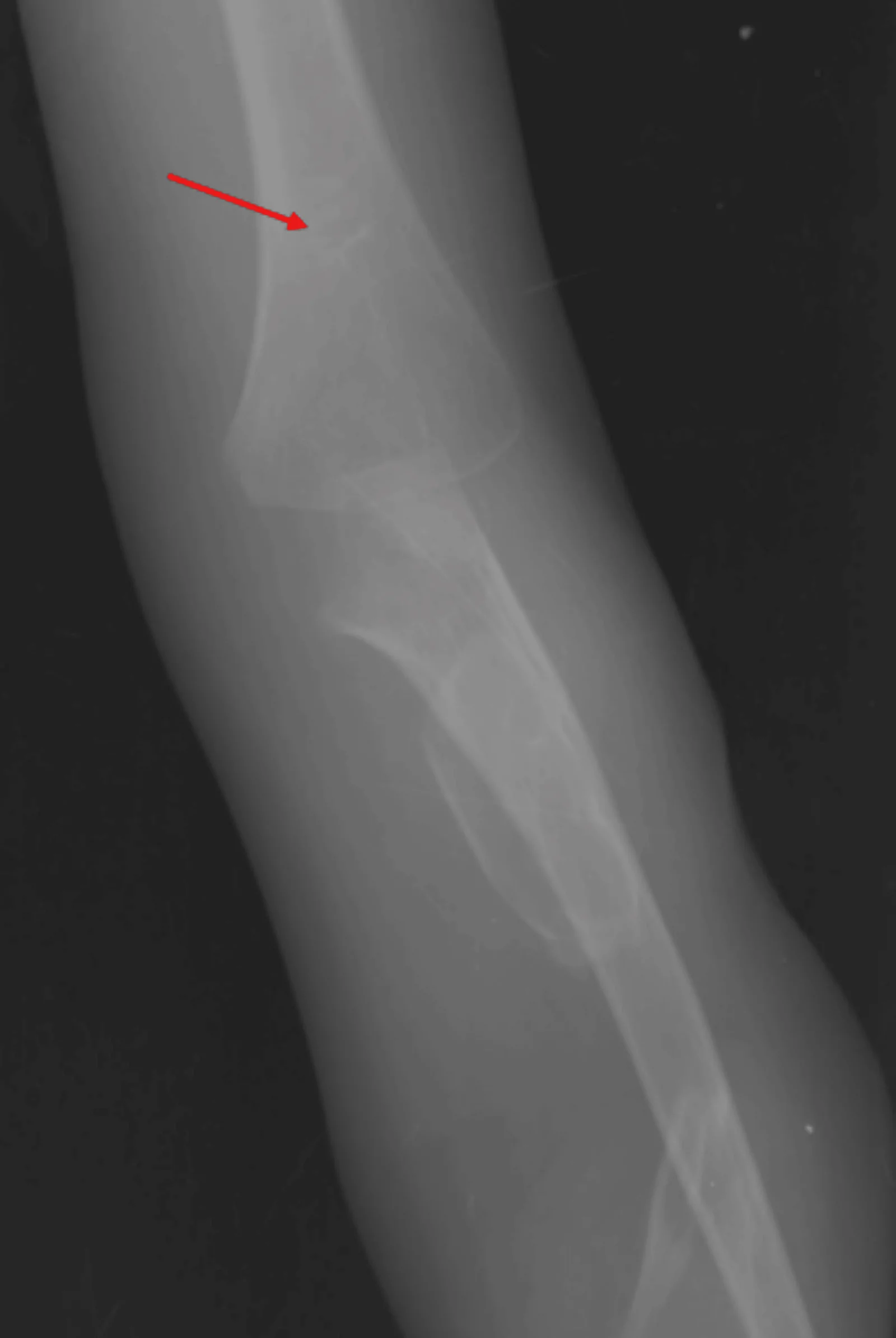
Lateral radiograph of the left elbow and forearm demonstrating a distinct, well-defined secondary osteolytic lesion localized within the medial humeral condyle (red arrow).

Given the progressive multisystem disease, extensive investigations were undertaken to exclude infectious, autoimmune, malignant, and hyperinflammatory conditions. Serological testing for human immunodeficiency virus (HIV), cytomegalovirus (CMV), parvovirus B19, and EBV was negative. Bone marrow aspiration demonstrated reactive marrow without evidence of tumor infiltration. Given the tuberculosis-endemic setting and lymph node involvement, tuberculosis was investigated using gastric aspirate testing for *Mycobacterium tuberculosis*, a tuberculin skin test, and a QuantiFERON-TB Gold assay (QIAGEN N.V., Venlo, Netherlands). All results were negative.

Definitive diagnosis of RDD was confirmed upon lymph node biopsy, which demonstrated large histiocytes with abundant eosinophilic cytoplasm exhibiting emperipolesis. Immunohistochemistry showed strong expression of CD163, CD68, and S100 proteins and negativity for CD1a, supporting the diagnosis of RDD. A thoracoabdominal and pelvic CT scan was performed to assess the extent of disease. It revealed multisystem involvement, including multiple bilateral pulmonary nodules and micronodules, bilateral mediastinal, and hilar lymphadenopathies measuring 10-25 mm, and renal, hepatic, and additional osteolytic bone lesions.

Given the multisystem involvement with progressive osseous and visceral disease, systemic therapy adapted from the LCH-III (Third International Study for Langerhans Cell Histiocytosis) protocol [9] was initiated, consisting of oral prednisone at 40 mg/m²/day for four weeks followed by tapering over two weeks, and vinblastine at 6 mg/m² administered as an intravenous bolus on days 1, 8, 15, 22, 29, and 35. Response assessment at week seven showed marked clinical improvement, with complete resolution of pain and restoration of upper limb function, accompanied by a marked decrease in inflammatory markers and radiological regression of both osseous and visceral lesions. In light of this favorable clinical, biological, and radiological response, the patient proceeded to the maintenance phase of the LCH-III protocol at week eight, which consisted of intravenous vinblastine every three weeks, five-day pulses of oral prednisone every three weeks, and daily oral 6-mercaptopurine. The patient completed the planned 12-month treatment course without disease progression. At the last follow-up, seven years after diagnosis, the patient remained in complete remission, with persistence of a residual angular deformity of the affected upper limb as the only long-term sequela.

The laboratory, immunological, and serological findings of the two patients are summarized in Table 1. 

**Table 1:** Laboratory findings and differential diagnostic workup of the two pediatric patients with Rosai-Dorfman-Destombes disease ANA: antinuclear antibodies; anti-dsDNA: anti-double-stranded DNA; C3/C4: complement components 3 and 4; CMV: cytomegalovirus; EBV: Epstein-Barr virus; HBV: hepatitis B virus; HCV: hepatitis C virus; HIV: human immunodeficiency virus; NP: not performed; TST: tuberculin skin test

Laboratory parameter	Case 1	Case 2	Reference range
Hemoglobin (g/dL)	12	8	11.5–15.5
White blood cell count (x10^3^/µL)	7.5	20.5 (neutrophilic)	4.5–10
Platelet count (x10^3^/µL)	167	660	150–450
Peripheral blood smear	No blasts	No blasts	No blasts
Erythrocyte sedimentation rate (mm/h)	Normal	101 (1st hour)/120 (2nd hour)	<10
C-reactive protein (mg/L)	5	125	<20
Serum protein electrophoresis	Normal	Hypoalbuminemia, hyper α1, α2 and γ globulinemia	Normal pattern
Viral serologies (HIV, EBV, CMV, and hepatitis B and C)	Negative	Negative	Negative
Tuberculosis (gastric aspirate for M. tuberculosis, TST, QuantiFERON-TB Gold)	Negative	Negative	Negative
Autoimmune panel (ANA, anti-dsDNA, C3, C4)	Negative	Negative	Negative
Bone marrow examination	NP	Reactive, no tumor infiltration	Normal marrow

## Discussion

RDD remains a diagnostically challenging disorder because of its rarity, heterogeneous clinical spectrum, and nonspecific clinical and radiological manifestations, particularly in children [1]. With an estimated prevalence of approximately one in 200,000 and around 100 new cases diagnosed annually in the United States, it predominantly affects children and young adults, with a reported mean age at diagnosis of approximately 20 years, although cases may occur at any age [1-4]. A male predominance has been described, as well as a higher frequency among individuals of African descent [4,5]. In Morocco, pediatric RDD remains rarely reported, with only a limited number of published cases [5-8]. In our report, we present two pediatric cases involving a 20-month-old male and a five-year-old female, which aligns with the known presentation of RDD in early childhood described in the literature [2,7].

The pathogenesis of RDD remains incompletely understood and is probably heterogeneous [2]. Historically, RDD was considered a reactive inflammatory disorder, possibly triggered by infectious or immune-mediated mechanisms [1,2]. Several infectious agents, including EBV, HHV-6, CMV, parvovirus B19, and HIV, have been investigated, but no definitive causal relationship has been established [2]. More recent molecular studies have identified recurrent alterations involving the MAPK/ERK pathway, including *KRAS*, *NRAS*, *MAP2K1*, and *ARAF *mutations, supporting a clonal process in a subset of patients [10-12]. In our experience with Case 1, molecular analysis did not reveal mutations in the examined exons of *KRAS*, *NRAS*, or *BRAF* exon 15. This negative result does not exclude the diagnosis, as MAPK pathway alterations are detected only in a subset of RDD cases and *BRAF* V600E mutations are uncommon in this disease [10,11]. Importantly, establishing the baseline molecular status serves a crucial clinical purpose, as identifying targetable MAPK alterations is increasingly recommended to guide potential second-line targeted therapies (such as MEK inhibitors) in cases of disease progression or treatment failure [11].

RDD typically presents with painless, slowly progressive cervical lymphadenopathy, sometimes accompanied by fever or weight loss [1,2,13,14]. Cervical nodes are the most frequent site of involvement [14]. Our first patient illustrated this classical nodal form, presenting with isolated cervical lymphadenopathy without systemic or extranodal features. According to published series, extranodal involvement occurs in roughly 40-45% of patients, while multisystem disease is seen in about 19% of cases [1,2]. Our second patient presented with this severe form. Initially misdiagnosed as osteomyelitis due to painful forearm bone lesions, he rapidly progressed to multisystem disease with axillary lymphadenopathy, respiratory distress, and pulmonary, hepatic, renal, and skeletal lesions. While rare, such aggressive presentations highlight the marked clinical heterogeneity of RDD in children [14]. These two cases reflect the broad spectrum of pediatric RDD, ranging from a benign, self-limiting lymphadenopathy to a severe multisystem disease requiring prompt systemic therapy. They highlight the need to consider RDD in children presenting with persistent lymphadenopathy or atypical osteolytic bone lesions.

Laboratory abnormalities in RDD are non-specific and primarily reflect the extent of underlying inflammation. Recommended initial evaluation includes a complete blood count, inflammatory markers (ESR and CRP), liver and renal function tests, and serum protein electrophoresis [2]. In our two patients, biological findings closely mirrored clinical severity. The first patient had entirely normal laboratory results, consistent with localized nodal disease. Conversely, the second patient presented with a marked inflammatory syndrome, including elevated ESR and CRP, microcytic anemia, and polyclonal hypergammaglobulinemia. Alternative infectious, autoimmune, and malignant diagnoses were systematically investigated and excluded. In particular, tuberculosis was thoroughly investigated and ruled out because of its high endemicity in Morocco and frequent presentation as chronic lymphadenopathy in children. Bone marrow aspiration was performed only in the second patient due to severe multisystem involvement and hematologic abnormalities; it showed reactive marrow with no evidence of tumor infiltration. This approach aligns with current guidelines recommending bone marrow examination only in patients with unexplained cytopenias or peripheral blood abnormalities [2].

Imaging plays a central role in the initial evaluation, staging, and follow-up of RDD [1]. In pediatric patients, ultrasonography of the neck and abdomen often serves as a first-line modality, whereas CT or magnetic resonance imaging (MRI) provides a detailed assessment of disease extent and extranodal involvement. Whole-body MRI can be valuable in children to avoid ionizing radiation, though limited availability and the need for sedation often constrain its use [2,13]. While 18F-fluorodeoxyglucose positron emission tomography/CT (FDG-PET/CT) is sensitive for identifying avid lesions and assessing therapeutic response, its routine pediatric use must be balanced against radiation exposure [2,13,15]. In our two clinical observations, ultrasonography was initially performed to characterize lymph node enlargement, while CT provided comprehensive staging. In the second patient, CT imaging proved essential in identifying the full extent of multisystem disease, revealing pulmonary nodules, mediastinal and hilar lymphadenopathies, hepatic and renal involvement, and widespread osteolytic bone lesions.

Definitive diagnosis of RDD relies on histopathological and immunohistochemical evaluation of biopsy specimens [1,2,13]. The hallmark histological feature is sinusoidal expansion by large histiocytes with abundant eosinophilic cytoplasm and prominent emperipolesis, the engulfment of intact lymphocytes within histiocytic cytoplasm, though this finding may be subtle in extranodal sites. On immunohistochemistry, RDD histiocytes characteristically express S100 protein and monocyte/macrophage markers (CD68, CD163), while lacking CD1a expression, thereby distinguishing RDD from Langerhans cell histiocytosis [1,2,13]. Additional markers such as OCT2 and Cyclin D1 can further support the diagnosis in challenging cases [2]. In both of our patients, tissue biopsies demonstrated typical histopathological features with evident emperipolesis, along with the classic S100+, CD163+, CD68+, and CD1a− immunophenotype. The second case underscores a major diagnostic pitfall; the initial bone biopsy in this infant was interpreted as a reactive histiocytic response secondary to osteomyelitis. The correct diagnosis of RDD was established only after a subsequent lymph node biopsy revealed classic emperipolesis and a confirmatory immunohistochemical profile [7]. This experience highlights the critical importance of clinicopathologic reassessment and repeat tissue biopsy when the clinical course is atypical or unresponsive to initial therapy.

There is no universally standardized treatment strategy for RDD, and management must be tailored to clinical symptoms, disease extent, organ involvement, and rate of progression [1,2,4,16]. As documented in consensus literature, observation is widely advocated for asymptomatic or mildly symptomatic nodal disease, uncomplicated cutaneous lesions, and fully resected solitary masses, as spontaneous regression occurs in a substantial proportion of patients [1,2,16]. This conservative strategy was successfully adopted in our first patient, who presented with localized cervical lymphadenopathy and no organ-threatening features. Progressive regression was documented during clinical and radiological follow-up, supporting active surveillance as a safe option in well-selected pediatric cases [14]. Systemic therapy, including corticosteroids and systemic chemotherapy, is reserved for symptomatic, refractory, progressive, or organ-threatening multisystem disease [2,4,16]. Corticosteroids are frequently used as first-line therapy to reduce tumor burden and systemic inflammation, though relapse upon tapering remains a risk. In severe pediatric presentations, vinca alkaloids (such as vinblastine) combined with corticosteroids have demonstrated efficacy [2,16]. This approach was applied in our second patient, who presented with aggressive multisystem extranodal disease, extensive osseous destruction, pulmonary nodules with respiratory distress, and hepatorenal involvement. Treatment with vinblastine and oral prednisone led to a rapid clinical, biological, and radiological response, achieving long-term complete remission despite a residual orthopedic deformity. Targeted agents, particularly MEK inhibitors, are emerging as valuable options for refractory cases with confirmed MAPK pathway mutations, though their pediatric use remains limited by molecular availability and long-term toxicity data [10-12].

The prognosis of RDD is generally favorable, particularly in isolated nodal or cutaneous forms, which frequently undergo spontaneous regression or respond well to conservative measures [1,2,4]. However, the clinical course remains unpredictable, and a subset of patients may experience progressive, relapsing, or organ-threatening disease [1]. Unfavorable prognostic factors include multifocal nodal disease, extensive extranodal involvement, and compromise of vital organs such as the liver, kidneys, lower respiratory tract, or central nervous system [1,2,4]. In our two observations, the overall disease evolution was favorable despite contrasting initial clinical presentations. The first patient, who presented with isolated nodal disease, experienced gradual regression under active surveillance. The second patient, despite presenting with severe, life-threatening multisystem disease, achieved complete remission following systemic chemotherapy, with a persistent angular bone deformity as the sole long-term sequela. In line with previous reports, our cases emphasize the necessity of prolonged clinical and radiological follow-up, as late relapses, disease progression, or long-term structural sequelae may occur [1,2].

## Conclusions

RDD disease in children shows a wide clinical spectrum, from simple cervical lymphadenopathy to severe multisystem involvement. Our cases highlight key diagnostic pitfalls, particularly bone lesions that can be mistaken for osteomyelitis. This shows the importance of re-evaluating the patient and performing repeat biopsies when initial treatments fail. Therapeutic management must be individualized based on disease extent and organ involvement. Active surveillance is suitable for uncomplicated nodal forms, whereas systemic chemotherapy combining vinblastine and corticosteroids can achieve durable remission in severe, progressive multisystem disease.
